# GrAPFI: predicting enzymatic function of proteins from domain similarity graphs

**DOI:** 10.1186/s12859-020-3460-7

**Published:** 2020-04-29

**Authors:** Bishnu Sarker, David W. Ritchie, Sabeur Aridhi

**Affiliations:** University of Lorraine, CNRS, Inria, LORIA, F-54000 Nancy, France

**Keywords:** Protein function annotation, Protein network, EC annotation, Label propagation, Domain similarity graph, GrAPFI, K-nearest neighbor

## Abstract

An amendment to this paper has been published and can be accessed via the original article.

## Background

Proteins are long sequences of amino acids that form the basis of life and plays vital role in all living organism through out the entire life-cycle. Proteins perform various functions in our body that needs to be understood to understand life, disease processes and guiding drug discovery efforts to combat the diseases. Due to the tremendous advancement in amino-acid sequencing technologies, it is now possible to sequence bulk amount of proteins in a rapid and affordable manner. Therefore, the number of protein sequences accumulating in public databases is rising at an unprecedented rate. This huge quantity of data calls for further exploitation and enrichment and it presents many challenges for biologists as well as computer scientists in annotating the functional properties of protein sequences. The UniProt knowledge base (UniProtKB) [1] is one of the most comprehensible protein databases as well as the largest public sequence database currently. It is divided into two main components: (i) the UniProtKB/Swiss-Prot database which contains protein sequences with reliable functional annotation of the protein sequences that has been reviewed by expert bio-curators, and (ii) the UniProtKB/TrEMBL database that stores unannotated and unreviewed sequences. Thus, for all proteins in UniProtKB, we have the primary amino acid sequence as well as some further information such as InterPro domain definitions which may have been identified from families of similar sequences or 3D protein structures.

The UniProt curators annotate UniProtKB/TrEMBL sequences using two complementary systems. The first, called UniRule, uses a large list of “if-then” rules. These rules have been generated manually, which is both a laborious and time consuming process. The rules in UniRule are generally very reliable but their coverage is low [2]. The second system is called Statistical Automatic Annotation System (SAAS), and was developed to support the labour-intensive UniRule system [3]. Automatic annotation rules are generated in SAAS using the annotations of the SwissProt sequences and a decision tree algorithm [4]. Other approaches exist for automatic protein function annotation. In particular, a number of techniques for predicting Enzyme Commission (EC) numbers that exploit protein structural similarities have been discussed in [5–7]. Many approaches based on sequence similarity have also been discussed in [8–11]. Several machine learning methods have also been studied extensively in [7, 12–20].

Recently, the notion of network science [21] has attracted great attention across many scientific communities. Network science has become a multidisciplinary area of research due to its ability to describe complex systems. It has found applications in many real-world scenarios from banking and the internet to modeling the human brain. Several approaches for annotating protein function have used network science and neighborhood based techniques to extract functional information from protein-protein interaction (PPI) networks and Gene Ontology terms [22–26]. A particular feature of biological networks is that they often require specialist biological knowledge to fully understand and exploit the network.

The following methods are widely used for predicting Enzyme Commission (EC) numbers using a variety of approaches based on machine learning, sequence encoding, functional domain similarity, and structural similarity. A deep learning based approach called DEEPre [17] predicts EC numbers putting together multiple tools and techniques including PSI-Blast [27], HMMER [28], Convolutional and Recurrent Neural Networks, and sequence encoding using position specific scoring matrix (PSSM) to perform dimensionality uniformization, feature selection, and classification model training. In recent years, deep learning has been applied in many computational biology and healthcare prediction tasks and achieved state-of-the-art performance. However, deep learning approaches can suffer from interpretability issues which is necessary in medical research and clinical decision-making [29].

EzyPred [19] is a k-nearest neighbor based method that adopts a top-down approach for predicting main class and sub-class of EC number. EzyPred works based on protein sequences only to perform the annotation task. It starts by predicting whether or not an input protein sequence is an enzyme. Then, EzyPred proceeds by predicting its main EC class and subclass. EzyPred uses pseudo amino acid composition [30] and functional encoding by exploiting functional and evolutionary information of proteins. Based on two features, EzyPred proposes a modified K-nearest neighbor classifier called OET-KNN (Optimized Evidence-Theoretic K-Nearest Neighbour). Although EzyPred performs well in terms of accuracy, it predicts only the first two digits of a four-digit EC number. Thus, its predictions are not very specific.

A machine learning based approach called SVM-Prot that uses support vector machine (SVM) for classification is proposed in [31–33]. And in 2016 [13], the performance of SVMProt is improved by adding two more classifiers: 1) K-Nearest Neighbor (KNN) and 2) Probabilistic Neural Networks (PNN). This approach uses important physico-chemical properties such as molecular weight, polarity, hydrophobicity, surface tension, charge, normalized van der Waals volume, polarizability, secondary structure, solvent accessibility, solubility, and the numbers of hydrogen bond donors and acceptors in side chain atoms to transform protein sequences into numerical feature representations.

A structure-based protein function annotation approach called *COFACTOR* is described in [6, 34]. The updated version of *COFACTOR* [35] combines information about protein structure and sequence homologs along with Protein-Protein Interaction (PPI) networks to form a hybrid model for jointly predicting GO terms, EC numbers, and ligand-binding.

EFICAz [9, 36, 37] presents a method for Enzyme Function Inference by Combined Approach. EFICAz combines predictions from four different methods using (i) recognition of functionally discriminating residues (FDRs) in enzyme families obtained by the authors’ “CHIEFc” procedure (Conservation-controlled HMM Iterative procedure for Enzyme Family classification), (ii) pairwise sequence comparison using a family-specific sequence identity threshold, (iii) recognition of FDRs in Multiple Pfam enzyme families, and (iv) recognition of multiple Prosite patterns of high specificity.

In ECPred [38], the authors describe a hierarchical prediction model. The model starts by predicting if a query sequence is an enzyme or non-enzyme. Once the query sequence is found to be an enzyme, ECPred predicts the main class to which the query sequence belongs. In the similar fashion, it follows the hierarchy of the EC numbering system to find the sub-class, sub-sub-class and sub-sub-sub-class. ECPred learns independent classifiers for 858 EC classes including 6 main classes, 55 subclass classes, 163 sub-subclass classes and 634 sub-sub-sub classes. The independent predictors that make up ECPred are SPMap, BLAST-kNN and Pepstats-SVM which are based on sub-sequences, sequence similarities, and the physico-chemical features of amino acids, respectively.

In this paper, we give a complete description of our novel graph based annotation approach(GrAPFI) [39], and we present an extended experimental analysis using test data from six popular reference proteomes from UniProtKB/SwissProt. GrAPFI builds network of proteins using domain and family information and performs neighborhood based label propagation for function annotation.

Although, similar to EZYPred [19] and SVM-Prot-KNN [13], GrAPFI is a neighborhood based classification technique, it uses different features and different inference mechanism that explores the network. GrAPFI uses InterPro signatures as domain information and label propagation over a weighted undirected graph built on proteins using their domain composition. Unlike COFACTOR [35], GrAPFI explores the functional domain architectures extracted from protein sequence instead of protein secondary structure and direct sequence homology. COFACTOR includes network information using PPI whereas GrAPFI builds network using jaccard similarity index between the domain composition of proteins. In contrast to ECPred and DEEPre [17] which are deep learning based method and learns class specific models for different classes, GrAPFI performs label propagation over weighted protein network to select the best EC annotation. ECPred learns 858 independent classifiers where as for GrAPFI, once the protein network is build, it’s ready for the inference of EC number using domain composition of the query protein.

We compare the performance of GrAPFI with the recently published ECPred approach. Along with ECPred, we also present the accuracy for DEEPre and SVMProt as representative examples of other state of the art EC number prediction approaches. Our analysis shows that GrAPFI gives better annotation performance than these earlier approaches.

## Results

### Data preparation

We have collected 262,564 proteins from the March 2018 release of Uniprot-KB/SwissProt [1] database using the following rules: (i) each protein must contain at least one InterPro signature and (ii) must be annotated with at least one EC annotation. After getting the protein data from each of the proteins, we have extracted InterPro domain composition and EC annotations. Then, we built the protein network as described in “EC annotation performance analysis” section. Each protein forms its own vertex. we did not preprocess training data to remove redundancy. Rather, while performing annotation, it ignores the same protein if it appears in the neighborhood. For example, for a query protein q, GrAPFI will collect the neighbors satisfying a maximum jaccard similarity score. When the maximum jaccard similarity is set to less than 1.0, GrAPFI omits the neighbors with exact same domain composition.

The training network covers 25 level-2, 31 level-3 and 408 level-4 EC classes from 41,618 oxidoreductases, 70,530 transferases, 100,027 hydrolases, 14,677 lyases, 25,551 isomerases, and 29,735 ligases which are linked using 10,866 InterPro signatures.

**Fig. 1:**
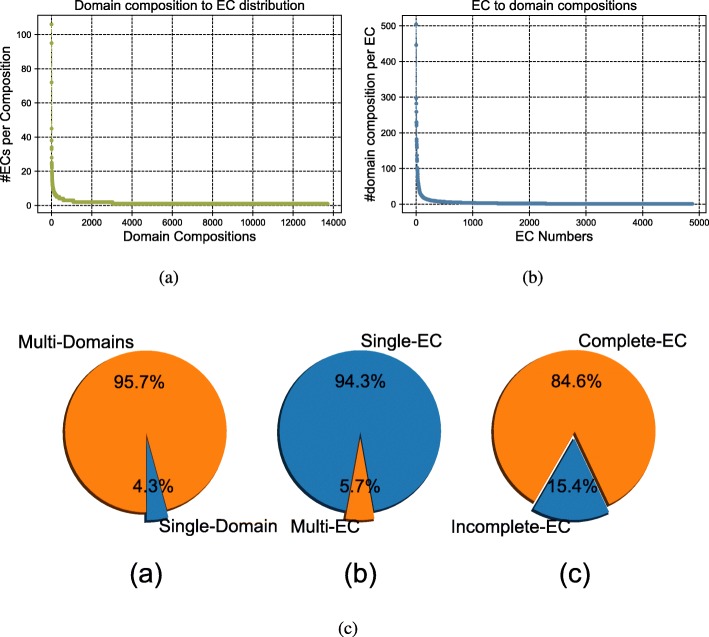
**a**: Distribution of EC numbers per domain composition, **b**: Distribution of domain compositions per EC number and **c**: training set statistics like **a** proportion of single-domain proteins, **b** single-EC proteins and **c** proteins with incomplete EC number

**Fig. 2:**
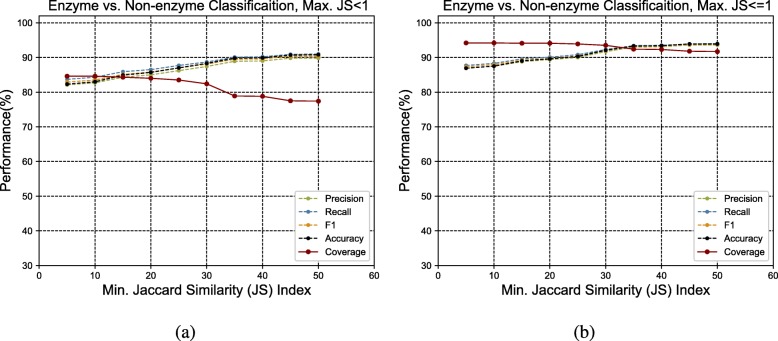
The precision, recall, F1, accuracy and coverage score for various minimum Jaccard similarity index for the *Enzyme vs. Non-enzyme* classification. **a** and **b** show the performance for upper similarity index of 1 and less than 1 respectively

**Fig. 3:**
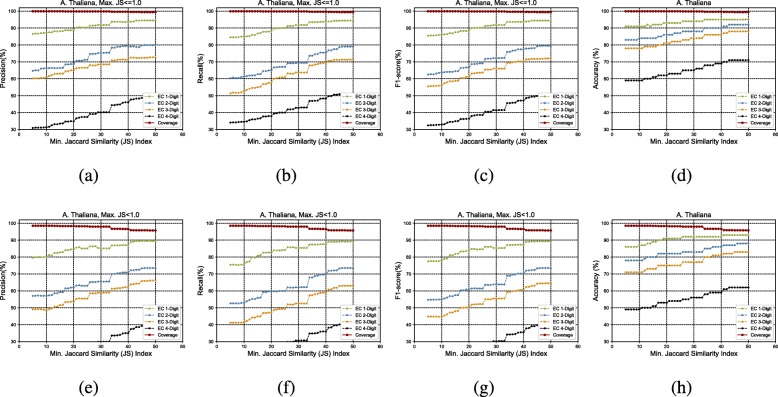
The accuracy and coverage for various minimum Jaccard similarity index for the *A. thaliana* reference proteome. **a** to**d** and **e** to **h** show the performance for upper similarity index of 1 and less than 1 respectively

In the training network, there are 1) 4.3% of the proteins are single-domain proteins i.e. proteins having only one domain in their domain composition (Fig. 1a, c), 2) 5.7% of the proteins have more than one EC numbers assined with them (Fig. 1b, c), and 3) Around 15% of the training nodes have incomplete EC annotations i.e. the EC numbers assigned with these proteins do not have all four digits. In the Fig. 1a, we show the distribution of EC numbers against domain composition. There are 13713 unique domain compositions in the training data. In the X-axis we put the domain compositions and along Y-axis we have the number of different EC annotations found for each domain composition sorted in descending order. It is evident from the figure that few of the domain compositions contain significantly higher number of EC numbers. For example, for some domain composition, there are more than 50 EC numbers found in the training data. We also show the distribution of domain compositions per EC number i.e. the different domain compositions found for each EC annotation shown in Fig. 1b. There are many cases when a higher number of domain compositions are mapped to a single EC. For example, in some cases, it is around 500 distinct domain compositions found against a single EC number. In essence, these two distributions reflect the dominance of many-to-many relationship between domain composition and EC annotation in the training data.

To valdiate GrAPFI, we used six popular reference proteomes from Uniprot-KB/SwissProt to as test set. The reference proteomes are the following: (i) *Rattus norvegicus* (UP000002494) containing 1,953 proteins, (ii) *Mus musculus* (UP000000589) containing 3,682 proteins, (ii) *Saccharomyces cerevisiae* (UP000002311) containing 1,581 proteins, (iv) *Homo sapiens* (UP000005640) containing 3,843 proteins, and (v) *Arabidopsis thaliana* (UP000006548) containing 5,352 proteins. (vi) *E. Coli* (UP000000625) containing 1465 proteins. For each of the reference proteomes, we collected the InterPro domains and EC labels from Uniprot-KB/Swissprot. We kept only the proteins which have at least one InterPro domain and are annotated with a single EC number.

To prepare the *COFACTOR* benchmark dataset, we used the 318 protein sequences published in [35], and we ran InterProScan [40] on these sequences to get their InterPro domain signatures. We only used IntePro domain signatures for the purpose of EC annotation.

### EC annotation performance analysis

To validate the annotation performance of GrAPFI, we computed the accuracy, Macro-precision, Macro-recall, and Macro-F1 score at different levels of EC number. For each query sequence, we picked the top ranked annotation only. The validation method we have used is similar to leave one out cross validation. For each proteomes, when annotating a protein, we have removed that protein from the training set so that a direct mapping is not present. we also present a 10-fold cross validation for enzyme vs. non-enzyme classification (Fig. 2a and b). The following formula (as used in [17, 18]) were used to compute the evaluation scores:

$$accuraccy(y, y')=\frac{1}{N}\sum_{i=0}^{N-1} 1(y_{i}=y'_{i}), $$

Here, y and *y*^′^ are the list of ground truths and predicted annotations. The accuracy is computed for each level of EC annotation. As the problem is a multi-class classification problem, we computed class-wise Macro-precision, Macro-recall, and Macro-F1 score as follows:

$$Macro-precision(y, y')=\frac{1}{|M|}\sum_{l \in M} precision(y_{l}, y'_{l}), $$

$$Macro-recall(y, y')=\frac{1}{|M|}\sum_{l \in M} recall(y_{l}, y'_{l}), $$

$$Macro-F1(y, y')=\frac{1}{|M|}\sum_{l \in M} F1\ measure(y_{l}, y'_{l}), $$

Here, *y*_*l*_ is the part of *y* with the label *l* and $y^{\prime }_{l}$ is the part of *y*^′^ with label *l*. And *M* is the set of classes. In general the precision, recall, and F1-Measure are computed as follows when two sets A and P are given:

$$precision=\frac{|A\cap P|}{|P|},$$

$$recall=\frac{|A \cup P|}{|A|},$$

$$F1-measure=\frac{2\times precision\times recall}{precision+recall}.$$

Here, A is the set of ground truths and P is the set of predictions. As EC numbers are hierarchical with 4 levels, we report level-wise precision, recall and F1-measure. Level-1 denotes main class, level-2 denotes sub-class, level-3 denotes sub-sub-class and level-4 denotes sub-sub-sub class. We also report coverage which is calculated according to *Coverage*=*M*/*T*, where T is the total number of proteins in the test set and M is the number of proteins for which at least one EC is predicted. For each query sequence, we consider the top-most annotation. For evaluation purposes, we split the 4-digit EC annotation into its constituent parts. Then, for level-1 we consider first digit, for level-2 we take first 2 digits, for level-3 we take first 3-digits and finally for level-4 we take all four digits together.

For the validation dataset, GrAPFI was run by setting different minimum jaccard similarity index ranging from 0.05 to 0.5, and setting an upper limit of the similarity to 1 or less than 1.

**Fig. 4:**
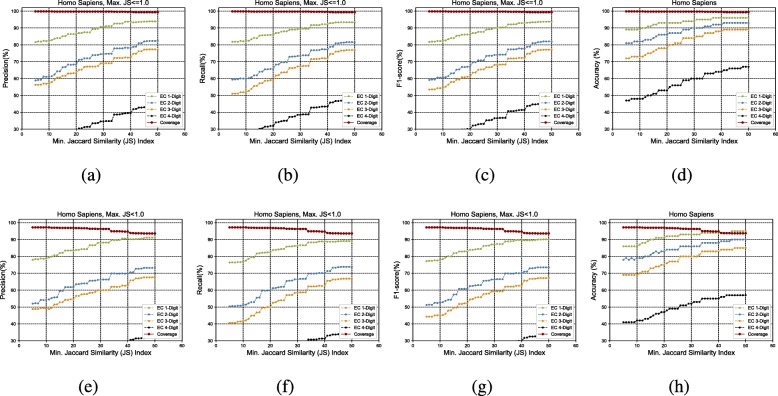
The accuracy and coverage for various minimum Jaccard similarity index for the *Homo Sapiens* reference proteome. **a** to **d** and **e** to **h** show the performance for upper similarity index of 1 and less than 1 respectively

**Fig. 5:**
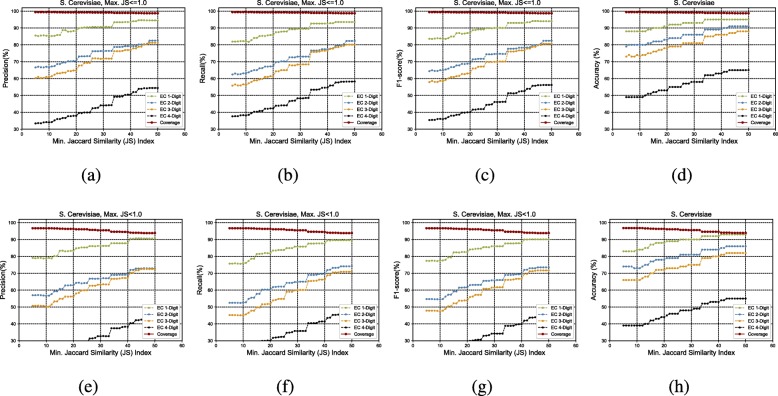
The accuracy and coverage for various minimum Jaccard similarity index for the *S. Cerevisiae* reference proteome. **a** to **d** and **e** to **h** show the performance for upper similarity index of 1 and less than 1 respectively

**Fig. 6:**
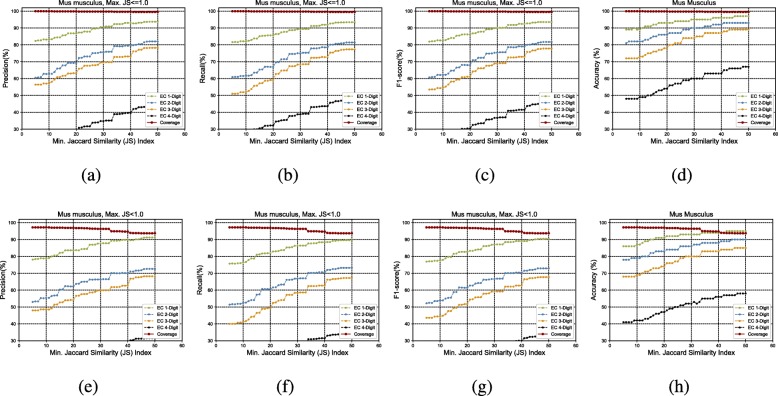
The accuracy and coverage for various minimum Jaccard similarity index for the *Mus Musculus* reference proteome. **a** to **d** and **e** to **h** show the performance for upper similarity index of 1 and less than 1 respectively

**Fig. 7:**
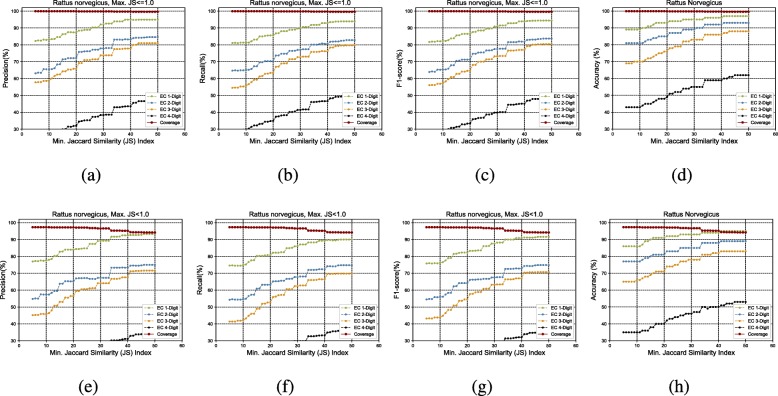
The accuracy and coverage for various minimum Jaccard similarity index for the *Rattus Norvegicus* reference proteome. **a** to **d** and **e** to **h** show the performance for upper similarity index of 1 and less than 1 respectively

**Fig. 8:**
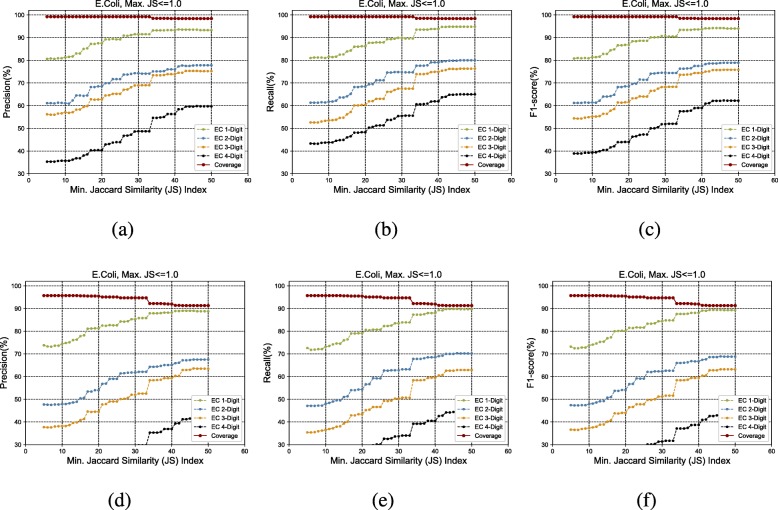
The accuracy and coverage for various minimum Jaccard similarity index for the *E.Coli* reference proteome. **a** to **c** and **d** to **f** show the performance for upper similarity index of 1 and less than 1 respectively

**Fig. 9:**
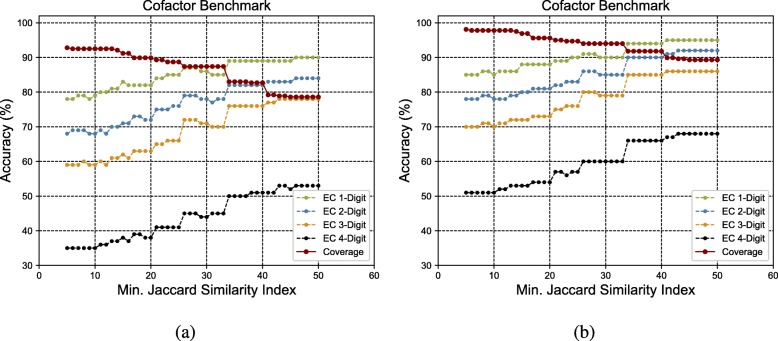
The accuracy and coverage for various minimum Jaccard similarity index for the *COFACTOR* benchmark proteins. **a** and **b** show the performance for upper similarity index of 1 and less than 1 respectively

Figure 3a to h show the GrAPFI performance for the reference proteome of *A. thaliana* for various min. Jaccard similarity indices. Similarly, Figs. 4, 5, 6, 7 and 8 show the performance of GrAPFI for the reference proteomes of *Homo sapiens*, *S. cerevisiae*, *Mus musculus*, *Rattus norvegicus and E. Coli*, respectively. In Fig. 9, we also show the performance of GrAPFI on *COFACTOR* benchmark dataset for various Jaccard domain similarity index ranging from 0.05 to 0.5, and setting an upper limit of the similarity to 1 and less than 1.

In these figures, we show the annotation accuracy (Y axis) against different Jaccard similarity thresholds (X axis) for the respective proteomes. We have considered similarity thresholds ranging from 0.05 to 0.5 as the annotation coverage falls significantly after 0.5. For each of the thresholds, we present the accuracy for EC-1, EC-2, EC-3 and EC-4 digit prediction shown in green, blue, orange and black color respectively. Along with accuracy, we also present the coverage of annotation (red curve). For each of the figures, we have two parts. The first part shows the accuracy and the coverage considering only the neighbors who have a Jaccard similarity of less than 1. The second part considers the Jaccard similarity of less than 1. It can be seen from these figures that GrAPFI performs very well for all of the cases with a good coverage.

**Fig. 10:**
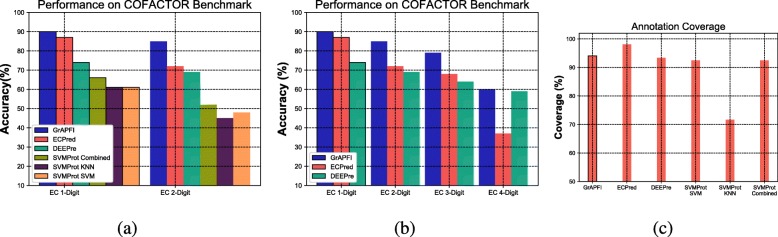
Part **a** shows a performance comparison of GrAPFI with SVMProt (SVM, KNN, and Mixed), DEEPre, and ECPred for 2-digit EC number predictions. Part **b** shows the accuracy comparison of GrAPFI with DEEPre and ECPred for all 4 level of EC prediction. Part **c** shows the coverage of the considered methods

To compare GrAPFI with other state of the art methods, we considered three machine learning based methods, namely ECPred [38], DEEPre [17], and SVMProt [13]. The performance is compared based on the *COFACTOR* [35] benchmark having 318 sequences. The SVMProt prediction results cover three different conditions: (i) using SVM only, (ii) using KNN only, and (iii) using SVM, KNN and PNN combined. Figure 10a shows the performance analysis for EC level-1 and EC level-2 prediction. The results presented here are achieved using a lower Jaccard similarity index of 0.3 and upper similarity index of 1.0. A much lower similarity threshold brings false positives that significantly reduce the accuracy. Based on the obtained results, a similarity index of 0.3 achieves a good trade-off between accuracy and coverage.

Because not all of the methods can make predictions for all four EC levels, we compared GrAPFI only with ECPred and DEEPre for 4-digit EC numbers as shown in Fig. 10b. In Fig. 10c, we show the annotation coverage of the methods considered here. It shows that ECPred has superior coverage compared to other methods. The reason GrAPFI fails to achieve highest coverage is due to the fact that it is a neighborhood based annotation method. GrAPFI performs label propagation by filtering out weakly linked neighbors determined by a minimum similarity threshold. Due to this filtering action, for few cases, GrAPFI fails to suggest any appropriate annotation for query proteins. This reduces the total annotation coverage. However, on the other hand, GrAPFI increases the accuracy by considering strongly linked neighbors. As shown in Fig. 10b and c, GrAPFI has better accuracy compared to ECPred and DEEPre, but it gives slightly less coverage than ECPred.

### Enzyme vs. non-enzyme classification

GrAPFI can be used in Enzyme vs. Non-enzyme classification task in a similar fashion as described in above section. However, the training graph must include non-enzyme proteins. To experiment with enzyme vs. non-enzyme classification, To evaluate the method, we have used a well defined dataset of enzyme and non-enzyme proteins curated from UniprotKB [1]. This dataset is called “NEW” and was constructed as described in [17]:

1. The SWISS-PROT (released on September 7, 2016) database was separated into enzymes and non-enzymes based on their annotation.
2. To guarantee uniqueness and correctness, enzyme sequences with more than one set of EC numbers or incomplete EC number annotation were excluded.
3. To avoid fragment data, enzyme sequences annotated with ’fragment’ or with less than 50 amino acids were excluded. Enzyme sequences with more than 5000 amino acids were also excluded.
4. Redundancy bias is removed using CD-HIT [41] with 40% similarity threshold to sift the raw dataset, resulting in 22,168 low-homology enzyme sequences.
5. To construct the non-enzyme part, 22,168 non-enzyme protein sequences were randomly collected from the SWISS-PROT (released on September 7, 2016) non-enzyme part, which were also subject to the above (ii–iv) steps. Thus the original dataset contains 22,168 enzymes and an equal number of non-enzymes.

The dataset contains the protein sequences along with their respective EC annotations. We have run InterProScan5 [40] to identify the domains contained in the sequences. Later, with the domain information, we have built the training graph. This graph contains 40040 proteins with 54% enzymes and 46% non-enzymes connected based on their domain composition.

To evaluate the annotation performance, we present 10-fold cross validation on the training graph and average macro-precision, macro-recall, macro-F1 scores are computed for various jaccard similarity indices. The result shows performance of enzyme vs. non-enzyme classification only. The experimental outcomes are shown in Fig. 2a and b.

It is evident from the experimental outcome that GrAPFI can distinguish enzyme and non-enzyme proteins with a a good score in all evaluation metrics. However, the coverage goes down as we move towards higher similarity thresholds. One of the things to be noted that considering exact similarity match does not change the performance significantly as can be seen in Fig. 2b.

## Discussions

Here, we explore new ways of connecting proteins. The proteins are connected based on domains that are potentially linked to the protein functions. This eventually means that GrAPFI is biologically meaningful approach. One of the major advantages of using GrAPFI to annotate proteins is that it produces explainable high quality annotations with a relatively simple annotation pipeline. The potential is evident from the experimental results. Although GrAPFI performs well, there are few drawbacks of using GrAPFI. For example, GrAPFI works on domain composition that can be achieved using another tool. GrAPFI can not be used with proteins without domain information. And also for the proteins with single domain, in most cases, GrAPFI fails to find appropriate annotation. The reason for this failure is that for a single domain protein, it is highly unlikely that there will be any high quality neighbors that can share annotations that eventually left the protein without any labels or wrong one. In any case, if GrAPFI fails to find an annotation, it is possible to identify the reason behind the failure and it restricts itself from predicting any annotation. This attitude reduces the false positives. However, from the experiment, it is evident that GrAPFI performs with high annotation coverage. Unlike other hierarchical classification models like ECPred [38] and DEEPre [17], GrAPFI does not learn model for every class. Instead, it builts a giant network of proteins and apply label propagation for each query proteins. The described approach could easily be distributed in order to handle large protein databases. The method is scalable for larger dataset using big data processing frameworks like Hadoop/Spark. We therefore aim to extent GrAPFI to use a distributed processing framework for the large scale annotation of the entire UniProtKB/TrEMBL database. Moreover, there is still scope of improvement specially for level-3 and level-4 predictions. As a future plan, we envision to improve the method for more precise predictions and also to apply the similar approach for protein function annotation using Gene Ontology Terms.

## Conclusion

In this paper, We have extended and validated GrAPFI [39], a novel network based approach for automatic protein function annotation using the domain composition of the proteins. Pairs of proteins in the network are linked based on their jaccard similarity coefficient using InterPro domain composition.

Our neighborhood based label propagation algorithm was applied to the network in order to propagate annotations from reviewed proteins to non-reviewed query proteins. This approach was validated using six popular reference proteomes from UniProtKB/SwissProt. We also compared GrAPFI results with those of ECPred, DEEPre, and SVMProt as examples of state of the art EC prediction approaches using the *COFACTOR* dataset. This comparison shows that GrAPFI achieves better accuracy and comparable or better coverage with respect to these earlier approaches.

## Methods

GrAPFI combines the notion of protein domain similarity with a graph neighborhood inference technique for automatic EC number annotation. More specifically, the functional annotations of reviewed proteins in SwissProt are used to predict those of non-reviewed proteins in TrEMBL using label propagation on a complex network representation of protein sequence data. The GrAPFI algorithm first constructs an undirected weighted graph of the proteins using the domain composition of the reviewed proteins. Then, given an non-reviewed protein, a label propagation algorithm is applied to the protein graph in order to infer appropriate annotations.

### Notation

In this section, we first present some definitions and notations used in the paper.

**Graph:** A graph is a collection of objects denoted as *G*=(*V*,*E*), where *V* is a set of vertices/nodes and *E*⊆*V*×*V* is a set of edges.

**Weighted Graph:** A weighted graph is a graph which is represented as a three tuple *G*=(*V*,*E*,*W*) where:

- *V* is a set of nodes,
- *E*⊆*V*×*V* is a set of edges,
- *W* is a weight matrix where each cell *W*_*uv*_ represents a numerical weight of the edge (*u*,*v*)⊆*E*.

**Labeled Graph:** A labeled graph is a graph which is represented as a four tuple *G*=(*V*,*E*,*L*,*I*) where:

- *V* is a set of nodes,
- *E*⊆*V*×*V* is a set of edges,
- *L* is a set of labels,
- *I*:*V*∪*E*→*L* is a labeling function.

**Directed Graph:** A Directed graph *G*=(*V*,*E*) is a collection of objects where *V* is a set of vertices/nodes and *E*⊆*V*×*V* is a set of edges with ordered pair of vertices (*u*,*v*) such that (*u*→*v*)∈*E*.

**Undirected Graph:** An undirected graph is a collection of objects denoted as *G*=(*V*,*E*), where *V* is a set of vertices/nodes and *E*⊆*V*×*V* is a set of edges with unordered vertices (*u*,*v*) such that if (*u*→*v*)∈*E* exists then (*v*→*u*)∈*E* must exists.

**Neighbors:** The neighbors of a node *u* are defined as *N*(*u*)={*v*|(*u*,*v*)∈*E*}.

**Degree:** The degree of a node in a graph is the number of edges which touch it. The degree of a node *u* in a graph *G* is denoted *deg*(*u*)=*N*(*u*).

**Average Degree:** The average degree of a graph *G*=(*V*,*E*) is a measure of how many edges are in the set *E* compared to number of vertices in the set *V*. The average degree of a graph *G*=(*V*,*E*) is defined by *Avgdeg*=2|*E*|/|*V*|.

### Graph construction

We present here a novel way of connecting protein sequences using their associated InterPro domains. Domains may be considered as natural building blocks of proteins. Due to evolution, protein domains may have gone through changes such as duplication, fusion, recombination to produce proteins with distinct structures and functions [42]. Here, each node of the graph represents a protein, while a link between two nodes means that the proteins exhibit a given level of domain similarity. Thus, each node *u* is identified by a set of labels *L*(*u*), has a set of neighbours *N*(*u*), and for every neighbour *v* it has an associated weight *W*_*u*,*v*_. The overall aim is to propagate labels (i.e. annotations) from nodes having labels to similar nodes that lack labels.

To illustrate the construction of the protein graph, let us consider five proteins with symbolic names *P*1,*P*2,*P*3,*P*4, and *P*5. Let us assume that these proteins are composed of domains *D*1=(*d*1,*d*2,*d*3,*d*4),*D*2=(*d*1,*d*3,*d*5),*D*3=(*d*1,*d*2,*d*10),*D*4=(*d*5,*d*6,*d*1), and *D*5=(*d*4,*d*1,*d*10,*d*40,*d*7,*d*9,*d*12,*d*52,*d*100), respectively.

Domain composition of a protein is the set of domains found in a protein sequence and considered irrespective of order of appearance in the sequence. For example the domain information in *D*1=(*d*1,*d*2,*d*3,*d*4) can be used in any other order *D*1=(*d*1,*d*4,*d*3,*d*2). Therefore, the composition is not strictly linear. The overlapping of domains are not considered as long as the overlapped domains has a new domain identification.

It is then evident that proteins *P*1 and *P*2 contain two domains *d*1 and *d*3 in common. Therefore, proteins *P*1 and *P*2 may be linked and the number of shared domains may serve as a link weight given by

$${} W_{P1,P2}=|(d1,d2,d3,d4)\cap (d1,d3,d5)|=|(d1,d3)|=2.$$

In a similar way, proteins *P*1 and *P*5 may be linked with a link weight of |(*d*1,*d*2,*d*3,*d*4)∩(*d*4,*d*1,*d*10,*d*40,*d*7,*d*9,*d*12,*d*52,*d*100)|=|(*d*1,*d*4)|=2. In both cases, the link weight is 2. However, the link weight computed in this way does not reflect the relative strength of the relationship among the proteins. More specifically, in the first case the two proteins have |(*d*1,*d*2,*d*3,*d*4)∪(*d*1,*d*3,*d*5)|=|(*d*1,*d*2,*d*3,*d*4,*d*5)|=5 different domains, of which two are shared. In the second case, there are |(*d*1,*d*2,*d*3,*d*4)∪(*d*4,*d*1,*d*10,*d*40,*d*7,*d*9,*d*12,*d*52,*d*100)|=11 different domains of which again two are shared. Although two domains are shared in each case, P1 is intuitively more aligned with P2 than P5. Therefore, instead of using the above raw similarity score, we used the Jaccard similarity index, or Jaccard similarity coefficient, to reflect better the similarity in composition. This is calculated as $\frac {|A \cap B|}{|A \cup B|}$, where A and B are the two sets of constituent domains. Using the Jaccard similarity index, the link weights for P1 and P2 are calculated as

$$\begin{array}{*{20}l}W_{P1,P2}&=\frac{|(d1,d2,d3,d4) \cap (d1,d3,d5)|}{|(d1,d2,d3,d4) \cup (d1,d3,d5)|}\\&=\frac{|(d1, d3)|}{|(d1,d2,d3,d4,d5)|}=\frac{2}{5} = 0.4.\end{array} $$

Similarly, for P1 and P5, the link weight is calculated as

$${\begin{aligned} W_{P1,P5}&= \frac{|(d1,d2,d3,d4)\cap (d4,d1,d10,d40, d7, d9, d12, d52,d100)|}{|(d1,d2,d3,d4)\cup (d4,d1,d10,d40, d7, d9, d12, d52,d100)|}\\&= \frac{2}{11} = 0.18. \end{aligned}} $$

Using the Jaccard similarity index, the final graph is built in two simple steps. In the first step, the data files that contain reviewed protein information are parsed to collect the constituent domains of each protein. If the training data contains only sequences, InterProScan [40, 43] is used to find the domains associated with each of the protein sequences. Then the graph is built using the domain composition of the proteins.

It is worth mentioning that the order of the domains is not maintained while computing jaccard similarity index. Domain composition for each protein contains the set of unique InterPro signatures found in the sequence.

### Enzyme commission numbers

Enzymes are usually labelled following the Enzyme Commission (EC) system [44], the widely used numerical enzyme classification scheme. The EC System assigns each enzyme a four digits number. This classification system has a hierarchical structure. The first level consists of the six main enzyme classes: (i) oxidoreductases, (ii) transferases, (iii) hydrolases, (iv) lyases, (v) isomerases and (vi) ligases, represented by the first digit. Each main class node further extends out several subclass nodes, specifying subclasses of the enzymes, represented by the second digit. Similarly, the third digit indicates the sub-subclass and the fourth digit denotes the sub-sub-subclasses. Let us consider as an example a Type II restriction enzyme, which is annotated as EC 3.1.21.4. The first digit, 3, denotes that it is a hydrolase. The second digit, 1, indicates that it acts on ester bonds. The third digit, 21, shows that it is an endodeoxyribonuclease producing 5-phosphomonoesters. The last digit, 4, specifies that it is a Type II site-specific deoxyribonuclease.

### Label propagation for protein function annotation

**Fig. 11:**
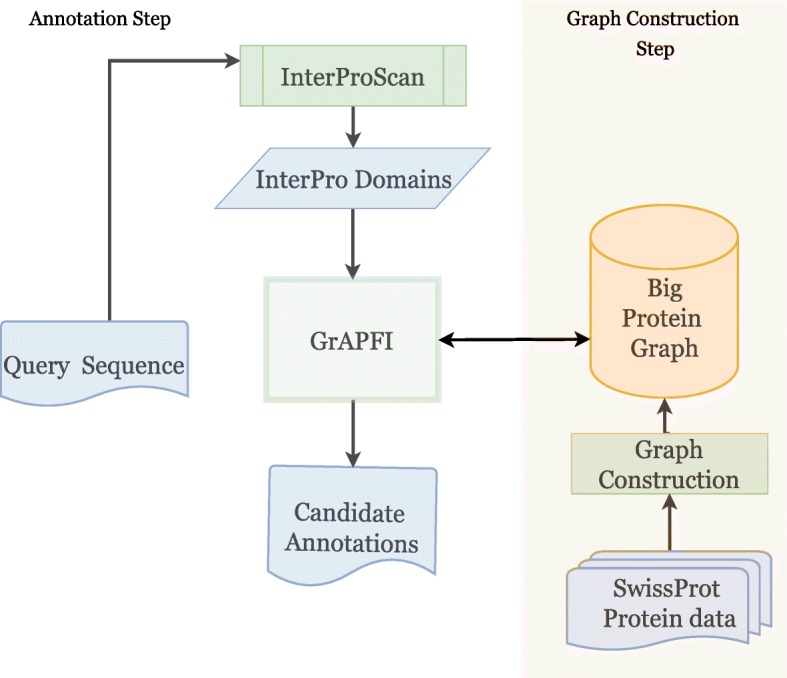
The annotation workflow used in GrAPFI. The right-hand portion of the workflow depicts the graph construction using reviewed proteins from the UniprotKB/Swissprot. The left part shows the annotation flow


After building the graph from the reviewed proteins, the graph is ready to be used for the function annotation of new protein sequences. A neighborhood based label propagation algorithm is designed to perform the annotation task. Given the constituent domains of an input protein sequence, all of its neighboring proteins and their annotations are retrieved from the graph. Once the neighbors have been obtained, the weighted frequency of the labels are computed using the following formula:

$$f^{i}_{u}=\frac{\sum_{v \in N(u)} W_{u,v} \delta(v^{i}, i) }{\sum_{v \in N(u)} W_{u,v}},$$

where $f^{i}_{u}$ is the weighted score of the candidate function *i* for the query protein *u*. And *δ*(*v*^*i*^,*i*) is 1 if the function *v*^*i*^ of the protein v is same as function i, otherwise, 0. The details of the label propagation algorithm is described in Algorithm 1. Overall, for a given input sequence, the annotation algorithm works according to the flow diagram shown in Fig. 11.

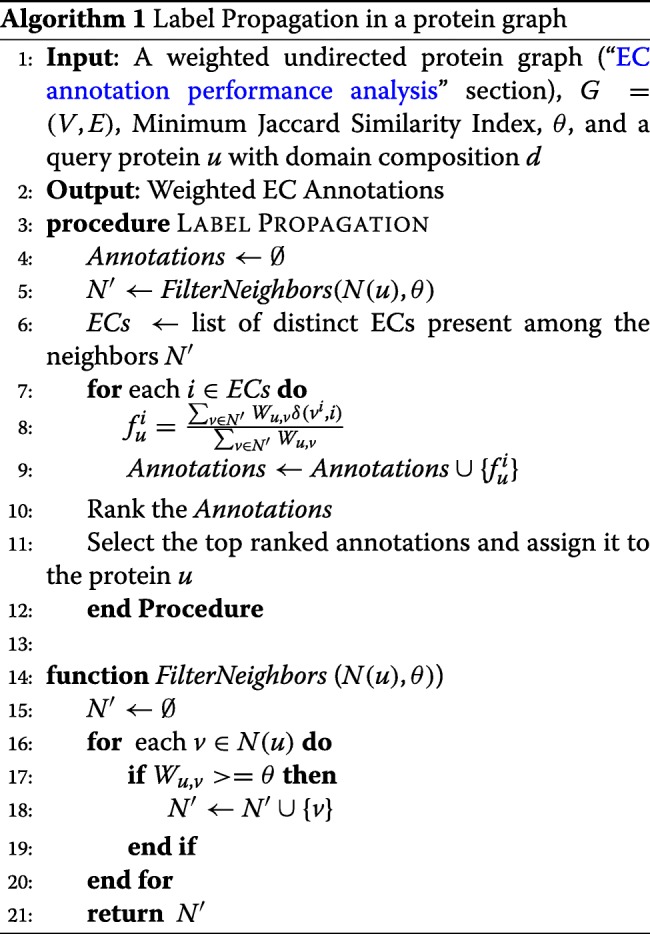


**Fig. 12:**
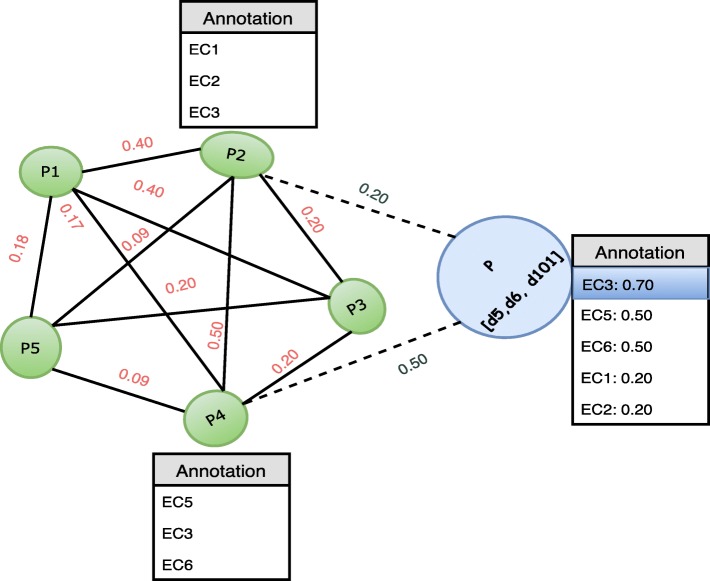
Example of EC annotation using label propagation

Let us consider a query protein *P* with a set of domains *D*=(*d*5,*d*6,*d*101). Our aim is to annotate this protein with an EC Number following the label propagation algorithm, as illustrated in Fig. 12. Based on the domain similarity, protein *P* will have connection with proteins *P*2 and *P*4 in the running example graph. The dotted lines show the links from *P* to *P*2 and *P*4 in the graph along with the associated weights. Therefore, the protein *P* will have *P*2 and *P*4 as it’s neighbors. After finding the neighbors, the functional annotations of all the neighbors are propagated along with the corresponding weights. All of the functional annotations are ranked based on their cumulative weights. The top ranked function is selected as the best functional annotation for protein *P*. In this example, the weighted annotations for *P* are *EC*3,*EC*5,*EC*6,*EC*1,*EC*2 with cumulative weights of 0.70, 0.50, 0.50, 0.20, and 0.20, respectively. Therefore, the functional annotation for the protein *P* is *EC*3 as it has the highest weight among the propagated labels. Clearly, it is possible to select more than one high scoring functional annotations if we wish to propose more than one candidate annotation. Furthermore, node neighbours could be selected in other ways to reflect the requirements of the problem at hand.

## Data Availability

The data and materials can be accessed here: https://gitlab.inria.fr/bsarker/bmc_grapfi.git

## Abbreviations

GrAPFI: Graph based automatic protein function inference EC: Enzyme commission GO: Gene ontology HMM: Hidden Markov model BLAST: Basic local alignment search tool PSI-BLAST: Position specific iterative basic local alignment search tool SAAS: Statistical automatic annotation system PPI: Protein protein interaction PSSM: Position specific scoring matrix OET-KNN: Optimized evidence theoretic K-nearest neighbor SVM: Support vector machine PNN: Probabilistic neural network KNN: K-nearest neighbor FDR: Functionally discriminating residues CHIEFc: Conservation-controlled HMM Iterative procedure for enzyme family classification
